# Virus infection mediates the effects of elevated CO_2_ on plants and vectors

**DOI:** 10.1038/srep22785

**Published:** 2016-03-04

**Authors:** Piotr Trębicki, Rebecca K. Vandegeer, Nilsa A. Bosque-Pérez, Kevin S. Powell, Beatriz Dader, Angela J. Freeman, Alan L. Yen, Glenn J. Fitzgerald, Jo E. Luck

**Affiliations:** 1 Biosciences Research, Department of Economic Development, (DED), 110 Natimuk Rd, Horsham, VIC, 3400, Australia; 2 Biosciences Research, DED, 5 Ring Road, La Trobe University, Bundoora, VIC, 3083, Australia; 3 Department of Plant, Soil and Entomological Sciences, University of Idaho, 875 Perimeter Drive MS 2339, Moscow, ID 83844-2339 USA; 4 Biosciences Research, DED, 124, Chiltern Valley Road, Rutherglen, VIC, 3685, Australia; 5 Institute of Agricultural Sciences-CSIC, Calle Serrano 115 dpdo., 28006, Madrid, Spain; 6 Agriculture Research, DED, 110 Natimuk Rd, Horsham, VIC, 3400, Australia; 7 Plant Biosecurity Cooperative Research Centre, LPO Box 5012, Bruce ACT, Australia

## Abstract

Atmospheric carbon dioxide (CO_2_) concentration has increased significantly and is projected to double by 2100. To increase current food production levels, understanding how pests and diseases respond to future climate driven by increasing CO_2_ is imperative. We investigated the effects of elevated CO_2_ (eCO_2_) on the interactions among wheat (cv. Yitpi), *Barley yellow dwarf virus* and an important pest and virus vector, the bird cherry-oat aphid (*Rhopalosiphum padi*), by examining aphid life history, feeding behavior and plant physiology and biochemistry. Our results showed for the first time that virus infection can mediate effects of eCO_2_ on plants and pathogen vectors. Changes in plant N concentration influenced aphid life history and behavior, and N concentration was affected by virus infection under eCO_2_. We observed a reduction in aphid population size and increased feeding damage on noninfected plants under eCO_2_ but no changes to population and feeding on virus-infected plants irrespective of CO_2_ treatment. We expect potentially lower future aphid populations on noninfected plants but no change or increased aphid populations on virus-infected plants therefore subsequent virus spread. Our findings underscore the complexity of interactions between plants, insects and viruses under future climate with implications for plant disease epidemiology and crop production.

Climate change is of global concern due to its predicted impacts on the environment and agriculture. Several atmospheric gases contribute to climate change including carbon dioxide (CO_2_). Since the beginning of the industrial revolution, the concentration of atmospheric CO_2_ has increased markedly and may more than double from its current level of about 400 μmol mol^−1^ to over 800 μmol mol^−1^ by the end of this century^1^. Numerous studies have addressed the different aspects of climate change in relation to plant physiology^2^. For example, elevated CO_2_ (eCO_2_) has positive effects on plant growth, expressed as higher yield, biomass, greater water-use efficiency, stimulation of higher rates of photosynthesis, changes to C:N ratios and improved light-use efficiency^3^,^4^,^5^.

Physiochemical changes to plants, mediated by eCO_2_ might have both direct and indirect effects on insect herbivores and on the epidemiology of pathogens transmitted by insects^6^,^7^. Studies that have investigated the influence of climate change on plant disease epidemiology indicate positive, negative or neutral effects^8^,^9^. Different climate factors including changes to temperature and CO_2_, drought, storm severity and rainfall, might affect the life cycle of plants, pathogens, and insect vectors and their dispersal as well as their interactions, and thus have the potential to alter the rate of infestation and pathogen spread^10^,^11^,^12^,^13^,^14^.

Changes to plant biochemistry occur when plants are exposed to higher CO_2_ levels, with a reduction of foliar nitrogen (N) concentration and a relative increase of carbon (C) (higher C:N ratios) due to the higher photosynthetic rates and growth^3^. Generally, many plant-chewing insects increase their feeding rates on plants grown under eCO_2_ to compensate for lower N levels in their host plant food source, but in many cases, the impact on insect growth and development is neutral or negligible and can be host plant specific^15^,^16^,^17^,^18^. Within the order Hemiptera, including aphids, there are reports suggesting an increase, decrease or no change to insect population levels when reared on plants subjected to eCO_2_^5^,^19^,^20^,^21^. Aphids are among the most important agricultural pests worldwide and are major vectors of plant viruses; therefore, understanding the impact of eCO_2_ on aphid population dynamics and interactions with pathogens is essential in order to predict epidemiology of plant diseases under future climate^6^.

According to models, cereal aphid populations are likely to be larger with increased CO_2_ concentration if N levels are high, but if higher temperature and eCO_2_ are combined, aphid populations may remain at levels similar to current ones^21^,^22^. Research investigating the bird cherry-oat aphid (*Rhopalosiphum padi*) using open top chambers suggests larger population size and fecundity under increased CO_2_ concentration^23^. In another study, *R. padi* abundance increased with higher CO_2_ concentration when reared independently, but decreased in the presence of the grain aphid *Sitobion avenae*^24^. Studies on five plant-aphid combinations revealed species-specific responses with, for example, *Myzus persicae* increasing and *Acyrthosiphon pisum* decreasing in population size when exposed to eCO_2_, and concluded that phloem-feeders may not be negatively affected by higher CO_2_ levels^20^.

*Rhopalosiphum padi* is one of the most economically important insect pests of wheat (*Triticum aestivum*), and is the main vector of *Barley yellow dwarf virus* (BYDV) which causes a disease that can reduce cereal grain quality and yield by over 70%^25^. Aphids exclusively transmit this virus during feeding^26^,^27^ and feeding time and virus concentration can affect transmission efficiency^28^,^29^,^30^. To date, there is a paucity of research addressing aphid/plant/pathogen interactions under eCO_2_, and in particular, limited research has been done on the impacts of eCO_2_ on virus-infected plants. Malmstrom and Field^31^ reported that growth of BYDV-infected oats (*Avena sativa*) under eCO_2_ can lead to an increase in biomass, potentially allowing an increase in aphid population size and reservoir for the virus. Trębicki *et al*.^13^ documented an increase of over 36% of BYDV titer in wheat plants grown under eCO_2_ compared to aCO_2_, and Nancarrow *et al*.^32^ showed that increased temperature resulted in an earlier and higher peak of BYDV titer in wheat.

There is considerable evidence showing that vector-borne pathogens (including BYDV) can modify their hosts making them more suitable to the insect vector, thus promoting acquisition, transmission and spread of the virus^30^,^33^,^34^,^35^. Additionally, pathogen-induced changes in infected insect vectors alters their behavior which can facilitate pathogen spread^36^,^37^,^38^,^39^.

The main objective of this study was to investigate the interactions between *R. padi*, BYDV and wheat (cv. Yitpi) under a future climate, taking into consideration current ambient and predicted elevated CO_2_ levels (aCO_2_ = 385 μmol mol^−1^ and eCO_2_ = 650 μmol mol^−1^). The development, fecundity and feeding behavior of *R. padi* (Fig. 1) on BYDV-infected and noninfected plants were examined under both ambient and elevated CO_2_ using controlled environment chambers. The effects of aCO_2_ and eCO_2_ on plant growth and biochemistry were also examined.

## Results

### *R. padi* development and fecundity

Two separate experiments were performed to study the development and fecundity of *R. padi* under ambient and eCO_2_. In the first experiment, we used noninfected plants, while BYDV-PAV-infected plants were used in the second experiment. *R. padi* development was unaffected by increased CO_2_ concentration on both noninfected and BYDV-infected wheat. The time between each instar, mean generation time and the period until *R. padi* reached maturity was similar for both CO_2_ treatments in each experiment (Table 1).

On noninfected plants, there was a significant negative effect of eCO_2_ on *R. padi* fecundity (Fig. 2a), which decreased by over 33% in average daily production of nymphs per adult compared to aCO_2_ (aCO_2_ = 5.08 and eCO_2_ = 3.37 nymphs, *P* < 0.001) (Fig. 3a). The average number of nymphs per adult within the time from birth to the onset of reproduction (Md) decreased by 26% under eCO_2_ and by 34% during the whole experimental period (M_12_) (Table 1). The intrinsic rate of natural increase (r_m_) and mean relative growth rate (RGR) also significantly decreased under eCO_2_ by around 10% (Table 1). Regardless of CO_2_ treatment, BYDV-infected wheat had no significant effect on aphid fecundity (Table 1, Figs 2b and 3b).

### *R. padi* probing behavior

To study *R. padi* feeding behaviour, we implemented the electrical penetration graph (EPG) system, a tool commonly used to determine the plant penetration activities by sup-sacking insects. Several measured parameters of *R. padi* probing behavior, recorded using the EPG system, were significantly affected when aphids were exposed to plants grown under eCO_2_ levels. In the first feeding behavior experiment on noninfected plants, a number of non-probing events, pathways, potential drops (cell punctures) and probes (C phases) significantly decreased (*P* < 0.05) for aphids reared on eCO_2_-grown wheat plants compared to plants grown at aCO_2_ (Fig. 4a and Table S1). The mean number of pathways decreased significantly by 36% under eCO_2_, while other probing activities (as listed above and in Fig. 4a) decreased between 41–44% (*P* < 0.05).

In the second feeding behavior experiment, BYDV-infected wheat plants were grown under aCO_2_ or eCO_2_ conditions and *R. padi* probing behavior was monitored. Parameters that were significantly higher in the noninfected plant experiment were, statistically non-significant on BYDV-infected plants regardless of CO_2_ levels(Fig. 4b and Table S2). However, xylem ingestion and penetration difficulties significantly increased on BYDV-infected plants grown under eCO_2_ (Fig. 4b, *P* < 0.05).

BYDV is a phloem-restricted virus, which requires aphid salivation into the phloem for transmission and phloem ingestion for acquisition. The duration of phloem ingestion (aphid feeding) on noninfected plants significantly increased by 34% (*P* = 0.007) under eCO_2_ compared to aCO_2_ conditions (Fig. 5a), but was unaffected on BYDV-infected plants (Fig. 5b). The mean duration (percentage of time) of *R. padi* probing activities devoted to phloem ingestion on eCO_2_-grown noninfected plants was 72%, compared to 54% on aCO_2_-grown plants. Additionally, on noninfected plants grown under eCO_2_, the duration of probes was significantly reduced by 38% (*P* = 0.01). No significant changes in the duration of xylem ingestion, phloem salivation or penetration difficulties were observed on noninfected plants grown under aCO_2_ and eCO_2_ (Fig. 5a). There was no significant difference between the time from first probe to phloem salivation between noninfected aCO_2_- and eCO_2_-grown wheat (aCO_2_ = 3433 s  ± 364 SEM, eCO_2_ = 4516 s  ± 596 SEM, *P* = 0.13) as well as other feeding parameters (Tables S1 and S2), suggesting a similar efficiency of BYDV transmission by aphids regardless of CO_2_ concentration. Similar to phloem ingestion, and in contrast to noninfected plants, other feeding behavior parameters were not significantly different in BYDV-infected plants grown under ambient and eCO_2_ (Fig. 5b).

EPG analysis can also reveal plant suitability and resistance mechanisms, either physical or biochemical. EPG variables including the time from the beginning of the recording to the first phloem feeding activity (E), time from the first probe to the first phloem feeding activity and number of probes before the first phloem feeding activity, might indicate increased plant resistance and aphid difficulties locating the phloem. During both experiments on noninfected and BYDV-infected plants, none of these variables were significantly different with increased CO_2_ (Tables S1 and S2). Increased plant resistance at the phloem level can manifest by an increased period of aphid salivation (total or mean duration of E1) or a number of single phloem salivation periods (E1). Again, none of these variables were significantly different in either experiment (Tables S1 and S2).

### Plant N concentration

To determine changes in N concentration (%), analysis was done on noninfected and BYDV-infected plants that were equivalent in terms of growing conditions, virus status and collection time to plants utilized on both the aphid performance and feeding behavior experiments. Elevated CO_2_ had a significant negative effect on the concentration of N and C:N ratio in above-ground biomass (leaves and stems combined) and roots of both noninfected and BYDV-infected plants (Fig. 6a,b and Fig. S3a,b). Under eCO_2_, N concentration of noninfected plants decreased by 42% in aboveground tissue (Fig. 6a, *P* < 0.001) and by 50% in roots (*P* < 0.001, Fig. 6b). For BYDV-infected plants, a decrease in N concentration was also recorded from plants grown under eCO_2_ (aboveground *P* < 0.001, roots *P* < 0.001) but the decrease was less pronounced than in noninfected plants. BYDV-infected plants had a 19% (*P* < 0.001) and a 31% (*P* < 0.001) decrease in N in aboveground plant parts and roots, respectively, when grown under eCO_2_, compared to aCO_2_-grown BYDV-infected plants (Fig. 6a,b). Under aCO_2_, virus status (i.e., presence or absence) did not affect the % N in aboveground or roots. However, under eCO_2_ conditions BYDV infection significantly increased N concentration by 28% (*P* < 0.001) in aboveground tissue and by 50% (*P* < 0.001) in roots, compared to noninfected plants. Increased CO_2_ concentration had a positive effect on growth of both noninfected and BYDV-infected plants (Fig. S4).

## Discussion

This is the first report that demonstrates that plant virus infection can mediate the effects of elevated CO_2_ on plants and insect vectors of pathogens. BYDV-infected plants from the eCO_2_ treatment contained significantly less N aboveground than both noninfected and infected plants from the aCO_2_ treatment but had 28% more N than noninfected plants from the eCO_2_ treatment. The fact that BYDV infection increased the relative aboveground N plant concentration under eCO_2_ (Fig. 6) (reducing the gap in N content relative to noninfected and BYDV-infected plants grown under aCO_2_), and that no negative effect of BYDV infection was observed on aphid performance and phloem ingestion (Table 1, Figs 2, 3, 4, 5), indicate that a major factor influencing aphids under eCO_2_ is plant nitrogen content. This indicates that virus infection can mediate the effects of eCO_2_ on wheat (cv. Yitpi) and as a consequence can improve plant suitability for the vector.

A decrease in plant N concentration may negatively influence the performance of *R. padi*, as shown by reduced fecundity and increased phloem ingestion in eCO_2_-grown noninfected plants (Table 1, Figs 2, 3, 4, 5), as it may be linked to changes in concentration of amino acids (N-containing compounds) that are essential for aphid nutrition^40^. Amino acid concentrations (absolute and relative to specific carbohydrates such as sucrose) are commonly reduced under eCO_2_, which may in turn influence aphid performance^24^,^41^. The use of leaf N as a proxy for nutritional quality for phloem-feeding insects is not as ideal as using phloem sucrose:amino acid ratios, and therefore, a more detailed analysis of phloem biochemistry of wheat under eCO_2_ would be justified^42^. However, aphids are known to exhibit a strong response to host plants with different nitrogen levels. For example, two cereal aphids, *R. padi* and *S. avenae* reared on four wheat cultivars at different N regimes, increased in adult weight, fecundity and longevity with increased N treatments^43^. Cotton aphid (*Aphis gossypii*) abundance was also positively correlated with plant nitrogen content^44^. In our study, the intrinsic rate of increase of *R. padi* on noninfected plants was significantly reduced under eCO_2_ by around 10%, which potentially can be attributed to N reduction in those plants. In another study, *R. padi*’s intrinsic rate of increase also decreased on nitrogen deficient seedlings^45^.

Generally, herbivorous insects will be worse off under future predicted CO_2_ concentrations, as lower nutritional plant quality will have an impact on the development of immature stages and result in increased mortality^16^. Although many sap-sucking insects have been shown to respond differently in their population levels under eCO_2_, and the range of responses can be attributed to host specificity and feeding requirements, no single factor so far influencing the response to eCO_2_ has been identified^15^,^16^,^17^,^18^,^19^,^20^ and it is very likely that diverse factors will influence the interactions differently. In our study, *R. padi* development time was not affected by increased CO_2_ concentration, but reduced fecundity and increased feeding rates were recorded on noninfected plants. We also showed a 42% reduction in N content in aboveground tissue in noninfected plants under eCO_2_ (Fig. 6a). Using open top chambers and three CO_2_ levels, population size and fecundity of *R. padi* were shown to increase under eCO_2_ but not nymphal development^23^. In another open top chamber experiment using double the ambient CO_2_ concentration, *R. padi* increased in abundance, when reared independently, but decreased in the presence of *S. avenae*^24^. In a greenhouse experiment, *R. padi* abundance increased on barley under elevated CO_2_ concentration but development and fecundity remained unchanged; additionally no significant changes to phloem amino acids were recorded^46^. Using growth chambers and pepper plants grown under ambient or eCO_2_, Dader *et al*.^5^, recorded 37% reduction in fecundity and 11% longer pre-reproductive period of *M. persicae* under eCO_2_. Additionally, significantly lower N content was recorded in pepper plants under eCO_2_, which was attributed to the reduced fecundity and increased pre-reproductive period of *M. persicae*^5^. Potential differences between our and other experiments might be attributed to the growing conditions, CO_2_ levels, fertilizer application, plant cultivar and age, aphid genotype among other factors.

To our knowledge, no previous study has examined the effect of eCO_2_ on virus-infected plants in relation to nitrogen concentration, and also examined nitrogen concentration thresholds within the plant tissue and their effect on aphid performance. Although eCO_2_ reduced the N concentration of BYDV-infected plants, the N concentration gap between BYDV-infected plants was less than the N concentration gap between noninfected plants (Fig. 6). No differences were seen in *R. padi* performance and feeding on BYDV-infected plants between the ambient and eCO_2_ treatments and this can be attributed to increased N content under eCO_2_ due to the virus presence.

Concentration and composition of free amino acids or other components can influence insect performance as shown in artificial diets^47^. Alternatively, it could be argued that reduced performance of *R. padi* on eCO_2_-grown plants could be affected by associated changes to plant resistance or to plant structure, such as an increase in cell wall thickness, which could contribute to insect feeding difficulties, reflected by a decrease in probing activity. However, with increased CO_2_, while significant changes to cell wall thickness have been recorded in rice, such changes have not been observed in wheat^48^. Different EPG variables can provide mechanistic explanations of the interactions between aphid and the plant and can highlight host plant suitability or resistance mechanisms^49^. The duration of aphid probing and other activities before the insect can locate the phloem, which is its main feeding source, can indicate increased or reduced plant resistance, which can be attributed to plant morphological or structural changes. Our study showed that the time from the beginning of the recording to the first phloem feeding, the time from the first probe to the first phloem feeding, and number of probes before the first phloem feeding were not different in both experiments (noninfected and BYDV-infected plants) (Tables S1 and S2). Therefore, despite the CO_2_ levels, the similar effectiveness in locating the phloem indicates that if any changes to plant morphology and structure took place, they were not a barrier-affecting aphid probing. Additionally, after locating the phloem (sieve elements) and prior to ingestion, sap-sucking insects including aphids, inject saliva (E1 waveform) to overcome potential plant resistance factors at the phloem level. The duration and frequency of the saliva secretion can indicate host plant suitability and it can define hosts and non-host plants^49^. Hence, we measured these variables but no significant differences were observed among treatments (Tables S1 and S2). As result, there was no indication of increased plant resistance to *R. padi* feeding on wheat grown under eCO_2_ (Figs 4 and 5, Tables S1 and S2). This suggests that the observed changes in aphid performance and feeding behavior are not caused by structural changes of plants but are more likely linked to plant biochemistry and nutritional quality (Fig. 6, Fig. S1).

Under ambient CO_2_ conditions, it has been shown that vector-borne plant pathogens can modify host phenotype and vector behavior aiding disease spread by increasing nutritional quality or attractiveness of infected plants to their vectors^33^,^36^,^38^,^50^,^51^. This is true for many persistently transmitted viruses including BYDV, but is also specific to particular aphid species, plant (including cultivars) and virus combinations, and severity of virus infection. Under aCO_2_ levels, when reared on plants infected with BYDV, aphids had a significantly shorter developmental time and/or increased fecundity compared to noninfected plants^35^,^50^,^52^. This may be caused by the increase in total amino acid content of plant tissue associated with BYDV infection^53^, but no conclusive studies have demonstrated this to be the case. We did not observe a shorter developmental period and increased fecundity of *R. padi* in BYDV-infected plants under aCO_2_ (Figs 2b and 3b). Differences between our findings and previous studies might be related to the experimental protocol or wheat variety utilized for the studies among other factors. In our study, C:N ratio decreased (Fig. S3) and N concentration increased (Fig. 6) in the eCO_2_-grown BYDV-infected plants, which might be associated with an increased level of amino acids, but additional studies would be required to assess this. Apart from the suggested alteration of nutritional quality of wheat, BYDV infection modifies the relative concentration of volatile organic compounds emitted by the plant, eliciting an aphid response, and increasing attractiveness and settling of nonviruliferous aphids^51^,^54^. Changes to *R. padi* behavior are also attributed to the presence or absence of BYDV within the vector. The ‘Vector Manipulation Hypothesis’ (VMH) by Ingwell *et al*.^36^, proposes that attraction of *R. padi* to either BYDV infected or noninfected wheat plants depends on whether the vector is carrying the virus, adding another layer to the already complex relationship between *R. padi*, wheat and BYDV, which can be further influenced by eCO_2_ or increased temperature. This complex interaction, which is often host, vector and virus-specific, has important implications for epidemiology and disease spread. Behavioral studies of virus-infected insects as influenced by CO_2_ levels are merited to determine if and how vector manipulation occurs under eCO_2_. Additionally, the effects of eCO_2_ on plant volatile organic compound profiles need to be assessed to understand the full impact of future climate on vector behavior.

As no evidence of increased plant resistance under eCO_2_ could be identified in this study, we predict that an eCO_2_ environment will not delay or reduce virus spread (Figs 4 and 5, Table S1 and S2). Additionally, under eCO_2_, no decrease in development and fecundity of *R. padi* on BYDV-infected plants was recorded, hence it is likely that eCO_2_ will not reduce the current levels of viruliferous aphids and virus spread. Moreover, increases in BYDV titer in wheat with increased temperature^32^ and CO_2_^13^ might intensify disease spread by increasing virus transmission and acquisition efficiency. Using oats and the BYDV pathosystem under aCO_2_ and eCO_2_, Malmstrom and Field^31^ concluded that increased persistence of BYDV-infected plants under eCO_2_ may alter disease epidemiology. Since the response of aphids to plants is species-specific and can be influenced differently by eCO_2_, the significance of non-crop hosts is also important if we are to understand aphid population dynamics and the initial introduction of the viruses into a crop.

Although other studies have investigated the effect of eCO_2_ on noninfected plant physiology and biochemistry to identify potential yield impacts, studies of interactions between eCO_2_ and virus-infected plants are scarce. Thus, understanding of these interactions and accurate predictions of virus epidemiology are required, and only then can we predict more realistic impacts on crop physiology and yield. In this first study, we move a step further, by including insect interactions, which are a vital component of virus epidemiology. Using noninfected, BYDV-infected wheat and coupling aphid performance with feeding behaviour studies (EPG) and plant biochemistry, provides mechanistic explanations of the interactions and their complexity. Further research is needed to understand the impact of a changing climate on the yield and quality of wheat cultivars overlayed by pests and diseases, which are important factors in agricultural production.

## Methods

### Insect and plant material

All *R. padi* used in this study were derived from a single parthenogenetic female obtained from vegetation located at the Grains Innovation Park (GIP) facility in Horsham, Australia. The clonal lineage was reared, in a constant-temperature growth chamber at 20 °C 14:10 D:L, for over 15 generations on wheat (cv. Yitpi) prior to the experiment. Five-week old wheat plants (cv. Yitpi) were used in all experiments. Plants were grown in 0.68 L plastic pots filled with potting mix containing slow release fertilizer, additional trace elements of iron and lime in temperature and CO_2_-controlled plant growth cabinets at 20 °C, and 14:10 D:L photoperiod (light intensity ca. 1000 μmol m^−2 ^s^−1^ at the top of the plant canopy, generated within each chamber by five 400 W high-pressure sodium and four 70 W incandescent globes) (Thermoline Scientific, TPG-1260). Potted plants were placed in trays and basal watered to maintain a standardised watering regime across all treatments. Growth cabinets were set at one of two CO_2_ levels (ambient: aCO_2_ = 385 μmol mol^−1^; or elevated: eCO_2_ = 650 μmol mol^−1^). On a weekly basis, CO_2_ concentrations and plants were alternated between the chambers to minimise any potential chamber effect.

### Virus source and inoculation

**Figure 1:**
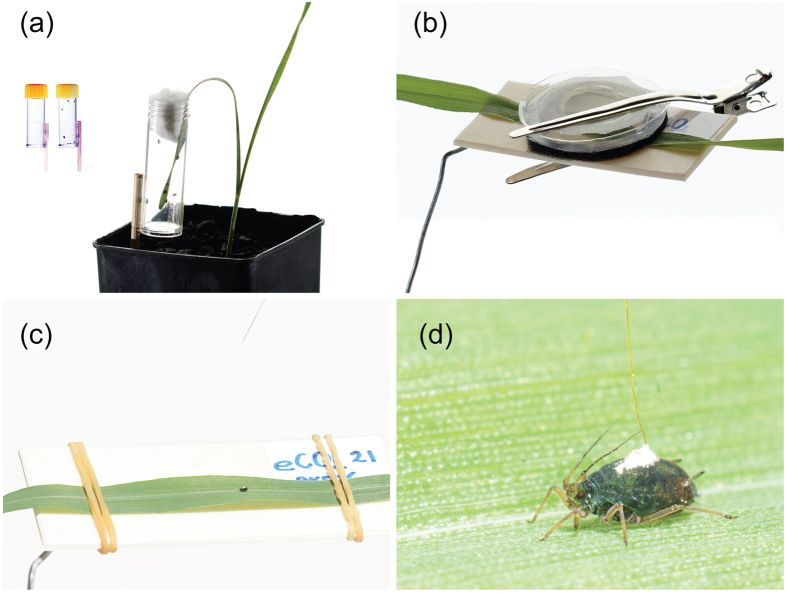
Illustration of (**a**) wheat inoculation with *Barley yellow dwarf virus*, where the tip of a leaf is inserted into a small tube containing 10 viruliferous adult *R. padi* and secured at the top with cotton wool to prevent aphid escape. All BYDV inoculations were performed using this method. (**b**) Clip cage used to study *R. padi* development and fecundity on noninfected and BYDV-infected plants under two CO_2_ levels. (**c**) During EPG, a leaf was placed on the support and secured with rubber bands to prevent plant movement. (**d**) Close up of *R. padi* probing on wheat, connected via silver glue and gold wire to the EPG insect electrode to record feeding behavior.

**Figure 2:**
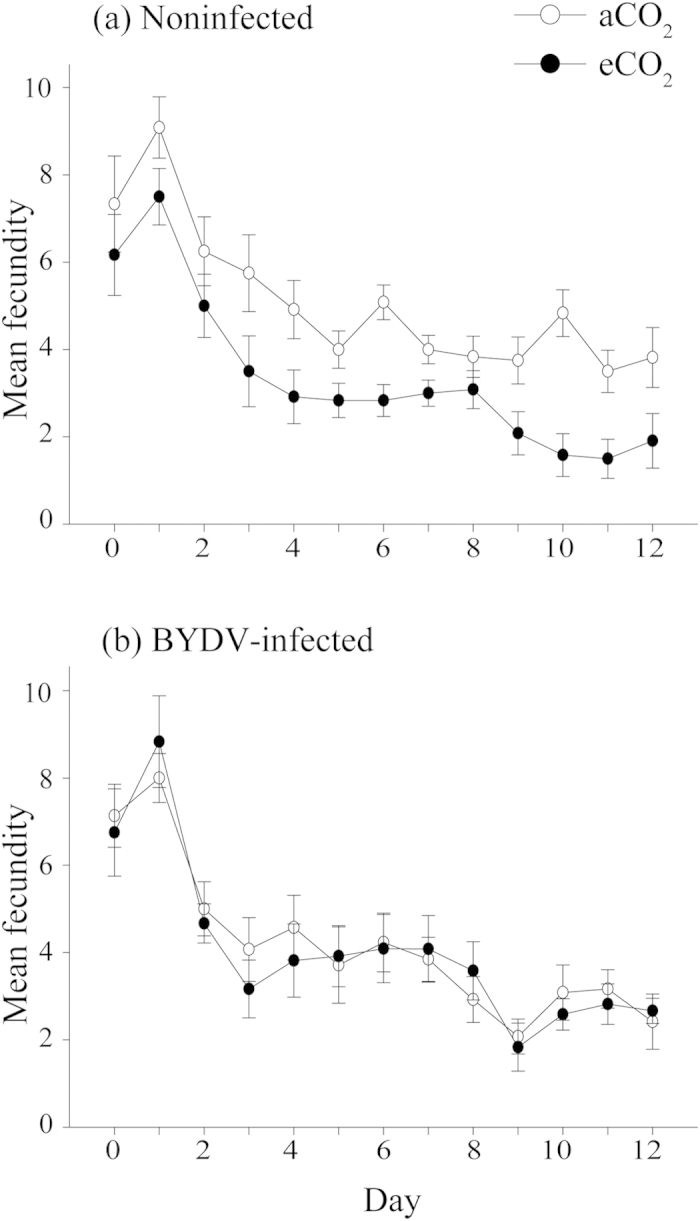
*R. padi* fecundity on (**a**) noninfected and (**b**) BYDV-infected wheat plants grown under ambient (aCO_2_; 385 μmol mol^−1^) or elevated CO_2_ (eCO_2_; 650 μmol mol^−1^). Measured by the daily count of newly emerged nymphs, where day 0 indicates the point when aphids first reached maturity. Error bars represent standard error (SEM). N = 17.

**Figure 3:**
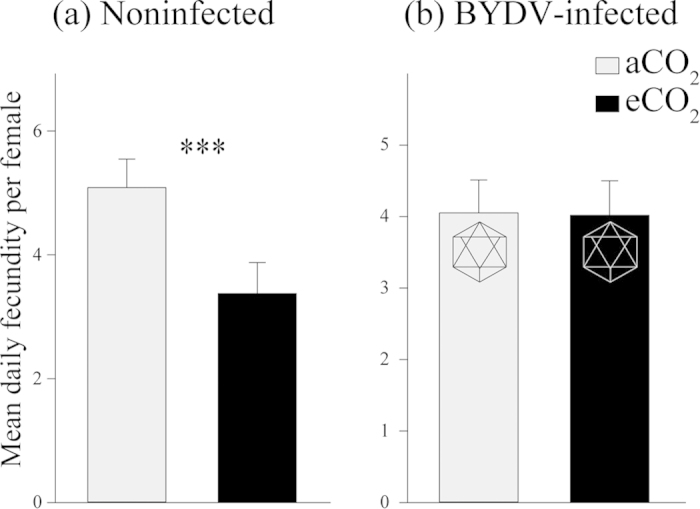
Mean daily fecundity per female recorded on wheat (**a**) noninfected and (**b**) BYDV-infected plants grown at ambient (385 μmol mol^−1^) or elevated (650 μmol mol^−1^) CO_2_ concentrations; ^* * *^ indicates significant difference at *P* < 0.001. Error bars represent standard error (SEM). Hexagon symbol indicates virus presence. N = 17.

**Figure 4:**
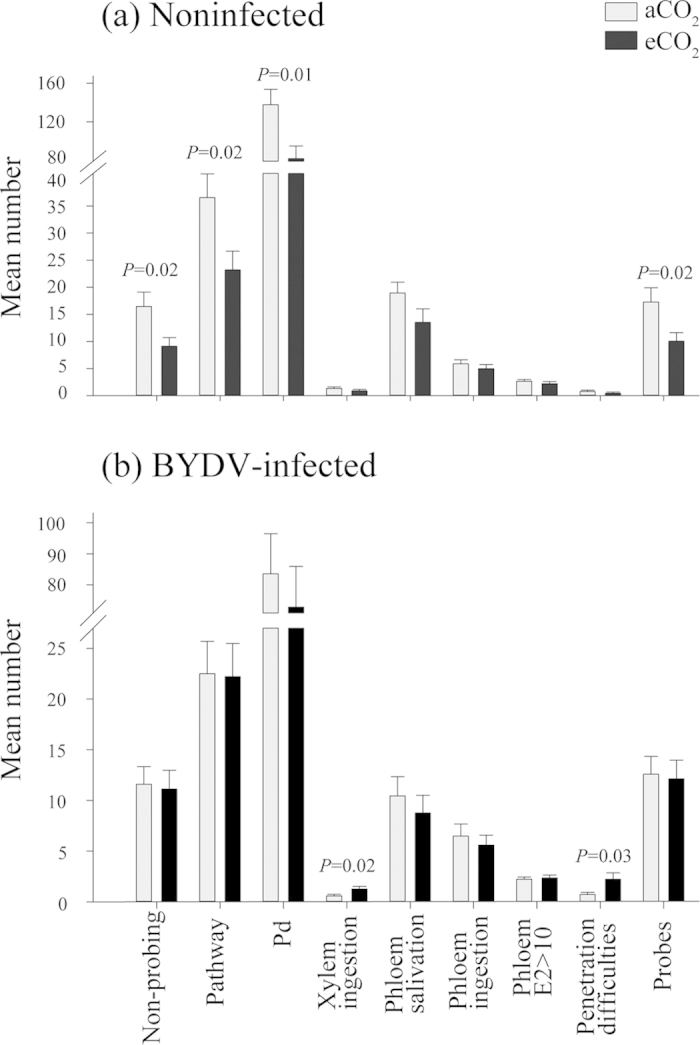
Number of *R. padi* probing activities on (**a**) noninfected plants and (**b**) BYDV-infected plants grown at ambient (385 μmol mol^−1^) or elevated (650 μmol mol^−1^) CO_2_ concentrations. Potential drop (Pd or intracellular penetration) indicates plant cell puncture, and Phloem E2 > 10 sustained phloem ingestion refers to phloem ingestion of ≥10 min. Where statistically significant, *P* values for each pair (aCO_2_, eCO_2_ treatments) are noted above the bars. Error bars represent standard error (SEM). Noninfected wheat N = 18, BYDV-infected wheat N = 22.

**Figure 5:**
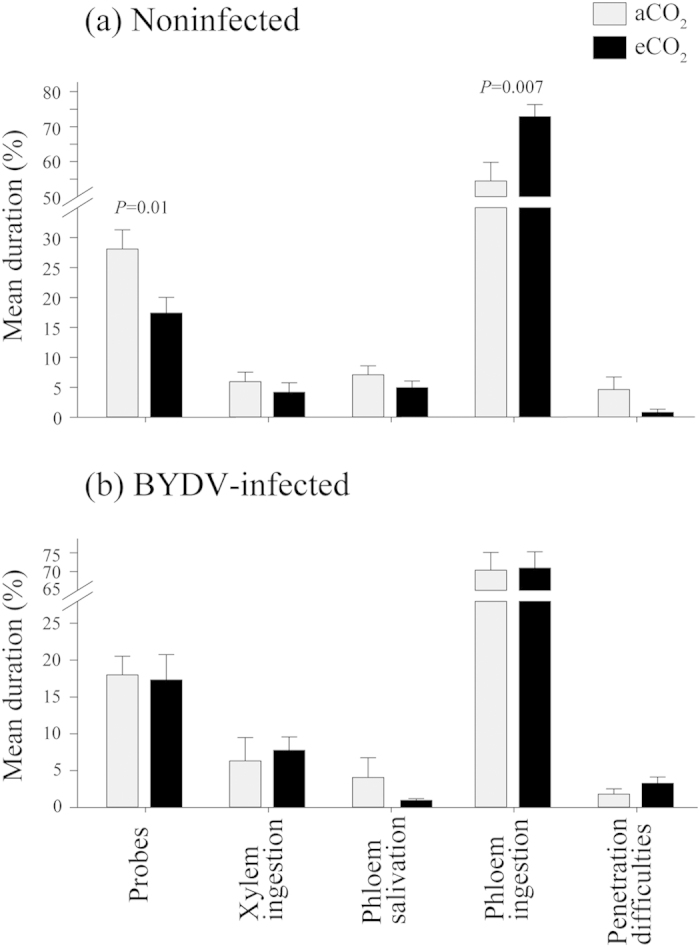
Percentage duration (time) of *R. padi* probing activities on (**a**) noninfected and (**b**) BYDV-infected wheat plants grown at ambient (385 μmol mol^−1^) or elevated (650 μmol mol^−1^) CO_2_ concentrations. Where statistically significant, *P* values for each pair (aCO_2_, eCO_2_ treatments) are noted above the bars. Error bars represent standard error (SEM). Noninfected wheat N = 18, BYDV-infected wheat N = 22.

**Figure 6:**
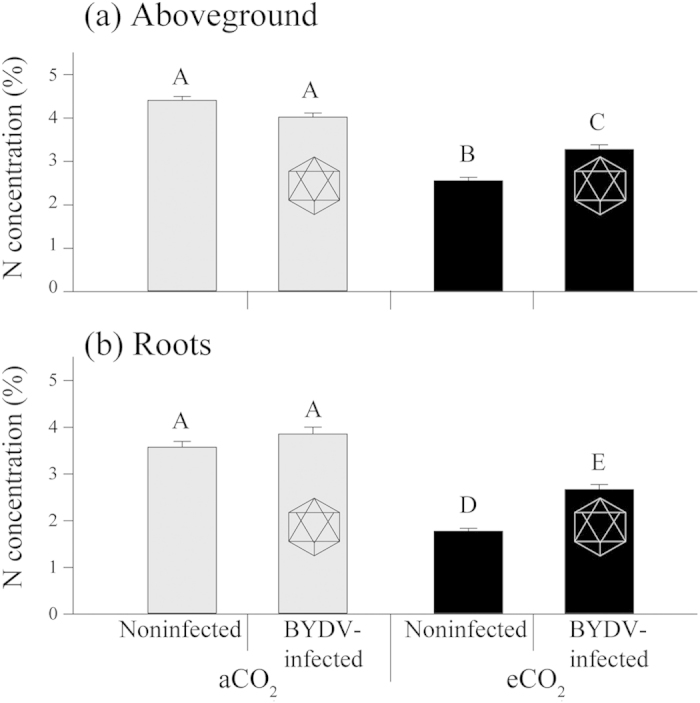
Nitrogen (N) concentration (%) of (**a**) aboveground plant parts and (**b**) roots of noninfected and BYDV-infected wheat plants grown under ambient (aCO_2_; 385 μmol mol^−1^) or elevated CO_2_ (eCO_2_; 650 μmol mol^−1^). Error bars represent standard error (SEM); different uppercase letters indicate significant differences between plant parts (aboveground and roots) and treatments (Tukey’s multiple range test, *P* < 0.05). Hexagon symbol indicates virus presence.

*Barley yellow dwarf virus*, PAV (BYDV-PAV) was used for all experiments to inoculate wheat plants in the virus positive treatment group. The PAV serotype was originally collected in Horsham, Australia from *A. sativa* plants located near the GIP Horsham premises. The virus was confirmed by PCR using PAV-specific primers and Tissue Blot Immunoassay (TBIA) methods^13^. To rule out multiple infection, PCR and TBIA tests for other species of BYDV or *Cereal yellow dwarf virus* (CYDV) were performed and found to be negative. BYDV-PAV was maintained at the DEPI facility on wheat (cv. Yitpi) in plant growth chambers (20 °C; 14D:10L). Subsequently, all experimental plants were also tested to confirm presence or absence of the virus using the TBIA method. Across all experiments, virus inoculation was standardised, using the same age source plants and aphid vectors. Eight to ten days after sowing, at the two-leaf stage, plants were inoculated with virus by exposure to 10 viruliferous aphids per plant. The tip of the leaf (approximately 4 cm long) of each plant was inserted into a small clear plastic tube containing the infected aphids and sealed with cotton wool (Fig. 1a). After 72 hours, all aphids were carefully removed.

### *R. padi* development and fecundity

To understand the effect of elevated CO_2_ on *R. padi* performance, two experiments were designed. In the first, we monitored the nymphal development and fecundity of *R. padi* on noninfected wheat plants grown under two CO_2_ conditions. In the second experiment, we used BYDV-infected plants, but otherwise all of the experimental conditions remained the same as in the first experiment. Both experiments were conducted in growth chambers, using the variety and growth conditions described above. A single young adult female was placed on the 3rd leaf of the main stem and housed in an individual clip cage (Fig. 1b). After four hours, the adult aphid was removed and all but one newly emerged nymph was left. To measure development, 17 insect replicates were assessed every 24 hours until adulthood. During each assessment, instar number were recorded and shed exuvia were removed. Once aphids reached adulthood, fecundity was monitored on 17 biological replicates (one plant and one insect each) by counting and removing nymphs every 24 hours for 12 days.

### *R. padi* feeding behavior

To understand the effect of elevated CO_2_ on aphid feeding behavior the electrical penetration graph (EPG)^55^,^56^ method was used. EPG is a commonly used method to study feeding behavior on many sap-sucking insects^38^,^49^,^57^,^58^. *R. padi* (1–4 day-old adults) was monitored first on noninfected wheat plants grown under elevated or ambient CO_2_ conditions (18 biological replicates, 16 h duration of each recording). Then feeding behavior was assessed on BYDV-infected plants grown under elevated or ambient CO_2_ conditions (22 biological replicates, 14 h duration of each recording). All experimental conditions remained the same across both experiments. EPG recordings were performed using the same settings, with plant voltage adjusted for each channel to ensure the first insect probe was always positive with a maximum amplitude of around +4 V. For each recording, the quality of silver glue connection between aphid and insect electrode was tested by using a calibration pulse after the first probe was initiated, and a good contact was determined by an output signal in the form of a square pulse. Probing by *R. padi* was monitored using an EPG Giga-8 amplifier (EPG-Systems, Wageningen, The Netherlands). All recordings were conducted within a Faraday cage housed inside a climate-controlled laboratory room (22 + 3 °C). EPG output was set to 50x gain and data was acquired at 100 Hz using a DATAQ Di710 A/D data acquisition USB device card (Dataq® instruments, Ohio, USA). EPG waveforms, which represent specific probing activity, were (Figs S1 and S2) analysed using Stylet+a software (EPG systems, Wageningen, The Netherlands). Since aphids are active when disturbed, each insect was transferred onto a vacuum device platform for tethering. Insects were attached to the electrode using a small droplet of water**-**based silver glue (EPG-Systems, Wageningen, The Netherlands) placed on the abdomen using a fine entomological pin (Fig. 1c,d). After 20 s, a second droplet of silver glue was added and a gold wire (12.5 μm diameter, 2 cm length) was placed in the glue and allowed to dry. The gold wire was attached by silver glue to a 0.2 mm diameter copper wire attached to a brass pin that was inserted into the input connector of the first-stage amplifier. Each wired aphid was left tethered for 1 h and then placed in the centre of the probing substrate.

### N analysis of plant tissue

To quantify potential effects of eCO_2_ on plant growth and biochemistry and the subsequent impacts on aphid performance and feeding behavior, another set of noninfected and BYDV-infected plants were grown at elevated or ambient CO_2_ conditions and analysed for total carbon (C) and nitrogen (N) concentration (%). Virus inoculation, growing medium, temperature and CO_2_ concentration were identical as for both previous experiments. Ten biological replicates each of five–week-old plants were harvested and the above-ground biomass (leaves and stems) and roots were dried at 65 °C for 48 h then finely ground (<0.5 mm) using a ball mill (Retsch MM 300). The percent of N and C concentration of aboveground biomass and roots was determined by the Dumas combustion method using a CHN analyser (CHN 2000; LECO, St Joseph, MI, USA) at the University of Melbourne, Creswick. The C:N ratio was calculated by dividing % C by % N for each plant tissue.

### Data analysis

To assess *R. padi* performance the intrinsic rate of natural increase (r_m_) was calculated^59^, using the equation r_m_ = 0.748^*^ (ln M_d_)/d, where 0.748 is a correction factor, d is the time from birth to the onset of reproduction and the M_d_ is the reproductive output per aphid that represents the duration of d that was used. Additionally, we calculated mean relative growth rate (RGR = r_m_/0.86), mean generation time (Td = d/0.738) and mean offspring number per female over the 12-day period (M_12_). Statistical analysis was performed on each individual insect EPG recording as well as on combined recordings for each treatment. Online resources were used to calculate EPG parameters (Tables S1 and S2)^60^. For aphids performance and feeding behaviour experiments, summary statistics and t-tests were performed using Microsoft excel and R statistical software, analysis of variance and Tukey’s HSD for multiple comparisons test was done using R statistical software for C and N between the treatments.

## Additional Information

**How to cite this article**: Trębicki, P. *et al*. Virus infection mediates the effects of elevated CO_2_ on plants and vectors. *Sci. Rep.* **6**, 22785; doi: 10.1038/srep22785 (2016).

## Supplementary Material

Supplementary Information

## Figures and Tables

**Table 1:** *R. padi* performance on noninfected and BYDV-infected wheat under ambient (aCO_2_; 385 μmol mol^−1^) or elevated CO_2_ (eCO_2_; 650 μmol mol^−1^)

	**Noninfected plants**			**BYDV-infected plants**		
	**aCO**_**2**_	**eCO**_**2**_	***P*** **value**	**aCO**_**2**_	**eCO**_**2**_	***P*** **value**
d	7.06 ± 0.17	7.05 ± 0.15	0.57	8.7 ± 0.25	8.7 ± 0.20	0.96
Md	45.9 ± 2.2	34.1 ± 4.5	0.0002	42.4 ± 3.6	42.6 ± 3.0	0.95
M_12_	65.8 ± 3.4	43.7 ± 3.1	0.0002	49.6 ± 3.9	51.2 ± 3.7	0.76
Td	9.3 ± 0.22	9.5 ± 0.57	0.57	11.2 ± 0.7	11.8 ± 0.3	0.43
r_m_	0.41 ± 0.01	0.37 ± 0.01	0.05	0.31 ± 0.01	0.32 ± 0.01	0.85
RGR	0.47 ± 0.02	0.43 ± 0.01	0.05	0.37 ± 0.01	0.37 ± 0.01	0.85

d is the time (days) from birth to the onset of reproduction, Md is the reproductive output per aphid that represents the duration of d; M_12_ is the mean offspring number per female over the 12 day period; Td is the mean generation time; r_m_ is the intrinsic rate of natural increase and RGR is the mean relative growth rate. ± sign indicates standard error of mean (SEM). N = 17.
