# Supplementary material for: Virus infection mediates the effects of elevated CO2 on plants and vectors
Source: Sci Rep. 2016 Mar 4;6:22785. doi: 10.1038/srep22785 (PMC4778167; doi:10.1038/srep22785)
Supplement: Supplementary Information [file srep22785-s1.pdf]

Supplementary Information

Virus infection mediates the effects of elevated CO_2 on plants and vectors

Piotr Trębicki[1], Rebecca K. Vandegeer[2], Nilsa A. Bosque-Pérez[3], Kevin S. Powell[4], Beatriz Dader[1,5], Angela J. Freeman[2], Alan L. Yen[2], Glenn J. Fitzgerald[6] and Jo E. Luck[7]

[1]Biosciences Research, Department of Economic Development, (DED), 110 Natimuk Rd, Horsham, VIC, 3400, Australia.

[2]Biosciences Research, DED, 5 Ring Road, La Trobe University, Bundoora, VIC, 3083, Australia.

[3]Department of Plant, Soil and Entomological Sciences, University of Idaho, 875 Perimeter Drive MS 2339, Moscow, ID 83844-2339 USA.

[4]Biosciences Research, DED, 124, Chiltern Valley Road, Rutherglen, VIC, 3685, Australia.

[5]Institute of Agricultural Sciences-CSIC. Calle Serrano 115 dpdo., 28006, Madrid, Spain.

[6]Agriculture Research, DED, 110 Natimuk Rd, Horsham, VIC, 3400, Australia.

[7]Plant Biosecurity Cooperative Research Centre, LPO Box 5012, Bruce ACT, Australia.

Corresponding author:

Piotr Trębicki, Telephone; +61 (0) 3 5362 2111, Fax; +61 (0) 3 5362 2187,

Email; piotr.trebicki@ecodev.vic.gov.au

Table S1. Measurements of *R. padi* feeding activity (±SEM) on noninfected wheat plants grown at ambient (aCO_2; 385 µmol mol-[1]) or elevated CO_2 (eCO_2; 650 µmol mol-[1]) concentrations in plant growth chambers.

	Noninfected plants						
Feeding parameters (EPG)	aCO_2			eCO_2			*P* value
Number of probes to the 1st E1	4.7	±	0.8	4.9	±	0.7	0.8
Total duration of F	4748.9	±	1122.9	1634.2	±	291.2	0.2
Mean duration of pd	5.2	±	0.2	5.7	±	0.4	0.3
Mean duration of F	3936.8	±	1114.1	896.1	±	138.2	0.2
Total duration of G	3868.2	±	804.2	4522.1	±	844.6	0.7
Mean duration of G	2766.7	±	711.4	2443.0	±	360.4	0.7
Number of single E1	12.4	±	1.5	8.1	±	1.8	0.1
Total duration of 1st E	245.1	±	63.6	1780.8	±	1369.0	0.3
Total duration the E1 followed by the first E2	97.3	±	18.2	91.7	±	10.3	0.8
Total duration of E	32044.8	±	2751.5	42199.4	±	1942.4	0.005
Total duration of E1	3595.7	±	720.3	2538.7	±	534.0	0.2
Total duration of E1 followed by E2	1365.6	±	334.5	1158.6	±	255.0	0.6
Total duration of single E1	2248.5	±	427.5	1397.8	±	341.1	0.1
Total duration of E2	28449.1	±	3020.1	39660.7	±	2335.1	0.006
Mean duration of E1	175.4	±	23.6	177.6	±	29.4	1.0
Mean duration of E2	7333.7	±	1508.9	11434.1	±	1644.1	0.07
Total duration of C	14174.9	±	1499.7	9209.2	±	1363.6	0.02
Total duration of np	5643.0	±	1404.0	2811.8	±	1043.6	0.1
Total duration of pd	711.5	±	86.0	453.1	±	70.8	0.03
Total probing time	51455.1	±	1411.8	53978.3	±	1095.1	0.2
Mean duration of np	495.7	±	219.7	316.2	±	81.7	0.5
Mean duration of C	406.2	±	26.6	402.8	±	21.5	0.9
Time from start of EPG to 1st E	3433.3	±	364.1	4516.0	±	596.7	0.1
Time from the beginning of that probe to 1st E	1984.7	±	370.7	2632.8	±	522.5	0.3
Time from start of EPG to 1st E2	6852.0	±	1884.0	6396.5	±	952.1	0.8
Time from the beginning of that probe to 1st E2	244.8	±	375.8	2365.1	±	340.7	0.8

Time and duration in seconds (E1, phloem salivation; E2, phloem ingestion; E, all phloem activity; pd, potential drops; G, xylem ingestion; F, derailed stylet mechanics; C, pathway; np, non-probing), N=18.

Table S2. Measurements of *R. padi* feeding activity (+SEM) on BYDV-infected wheat plants grown at ambient (aCO_2; 385 µmol mol-[1]) or elevated CO_2 (eCO_2; 650 µmol mol-[1]) in plant growth chambers.

	BYDV-infected plants		
Feeding parameters (EPG)	aCO_2	eCO_2	*P* value
Number of probes to the 1st E1	6.8 ± 0.9	6.4 ± 1.0	0.8
Total duration of F	1775.1 ± 324.8	2454.6 ± 360.8	0.4
Mean duration of pd	5.4 ± 0.2	5.2 ± 0.1	0.3
Mean duration of F	1105.4 ± 222.9	856.3 ± 120.8	0.6
Total duration of G	6247.4 ± 1421.6	5275.2 ± 857.0	0.8
Mean duration of G	5319.7 ± 1357.7	2992.5 ± 502.4	0.4
Number of single E1	3.5 ± 0.9	2.5 ± 0.8	0.4
Total duration of 1st E	7514.3 ± 3420.3	14897.0 ± 3997.8	0.2
Total duration the E1 followed by the first E2	1080.6 ± 1035.5	41.2 ± 3.6	0.3
Total duration of E	33604.5 ± 1990.2	34135.4 ± 2175.5	0.9
Total duration of E1	1639.8 ± 1049.4	416.5 ± 84.6	0.3
Total duration of E1 followed by E2	1377.9 ± 1041.2	223.9 ± 39.8	0.3
Total duration of single E1	322.7 ± 74.5	321.9 ± 58.4	1.0
Total duration of E2	31964.8 ± 2443.6	33718.9 ± 2246.4	0.6
Mean duration of E1	112.5 ± 57.0	48.1 ± 4.2	0.3
Mean duration of E2	12584.0 ± 3140.1	15821.1 ± 3659.8	0.5
Total duration of C	7876.4 ± 1070.0	7482.7 ± 1222.6	0.8
Total duration of np	5271.9 ± 1089.9	3375.1 ± 892.0	0.2
Total duration of pd	428.2 ± 60.6	368.8 ± 64.4	0.5
Total probing time	44862.2 ± 1103.6	46560.0 ± 895.1	0.2
Mean duration of np	802.7 ± 312.5	429.6 ± 149.4	0.3
Mean duration of C	367.9 ± 28.0	342.4 ± 23.5	0.5
Time from start of EPG to 1st E	4971.0 ± 550.0	5308.2 ± 564.88	0.6
Time from the beginning of that probe to 1st E	1529.1 ± 331.1	2231.95 ± 370	0.1
Time from start of EPG to 1st E2	8748.8 ± 2209.4	5974.1 ± 661.8	0.2
Time from the beginning of that probe to 1st E2	2733.2 ± 1055.4	2249.9 ± 360.8	0.6

Time and duration in seconds (E1, phloem salivation; E2, phloem ingestion; E, all phloem activity; pd, potential drops; G, xylem ingestion; F, derailed stylet mechanics; C, pathway; np, non-probing), N=22.

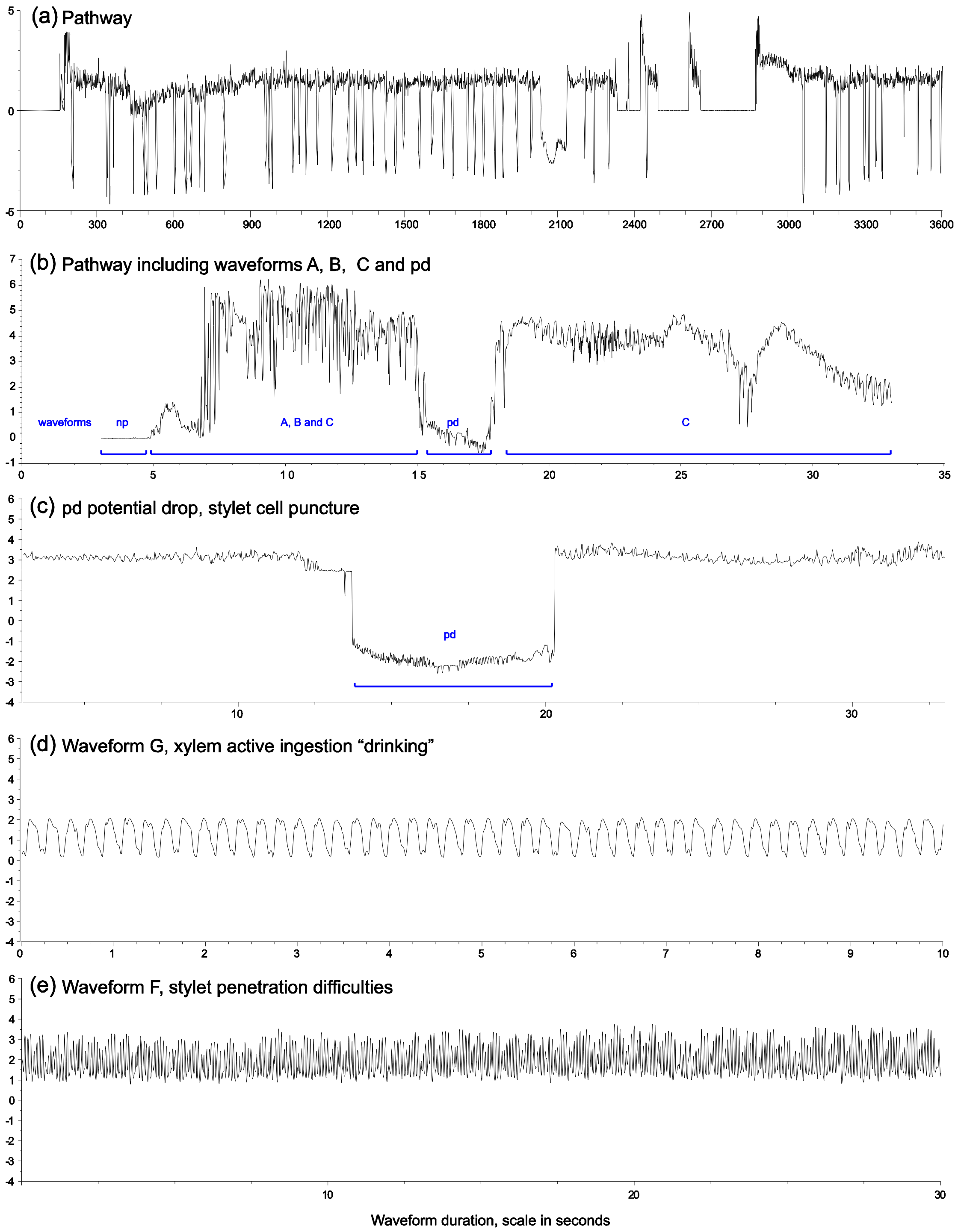

Fig. S1. Examples of waveforms representing different probing activities from EPG recordings of *R. padi* probing on wheat. (a) One hour duration from the beginning of the recording, (b) np, non-probing phase, stylet outside the plant tissue; C, probing phase; pd (potential drop), intracellular penetration, (c) pd, (d) G, xylem ingestion phase and (*E*) F, stylet penetration difficulties (derailed stylet mechanics). Y axis represents voltage level. X axis represents waveform duration (seconds).

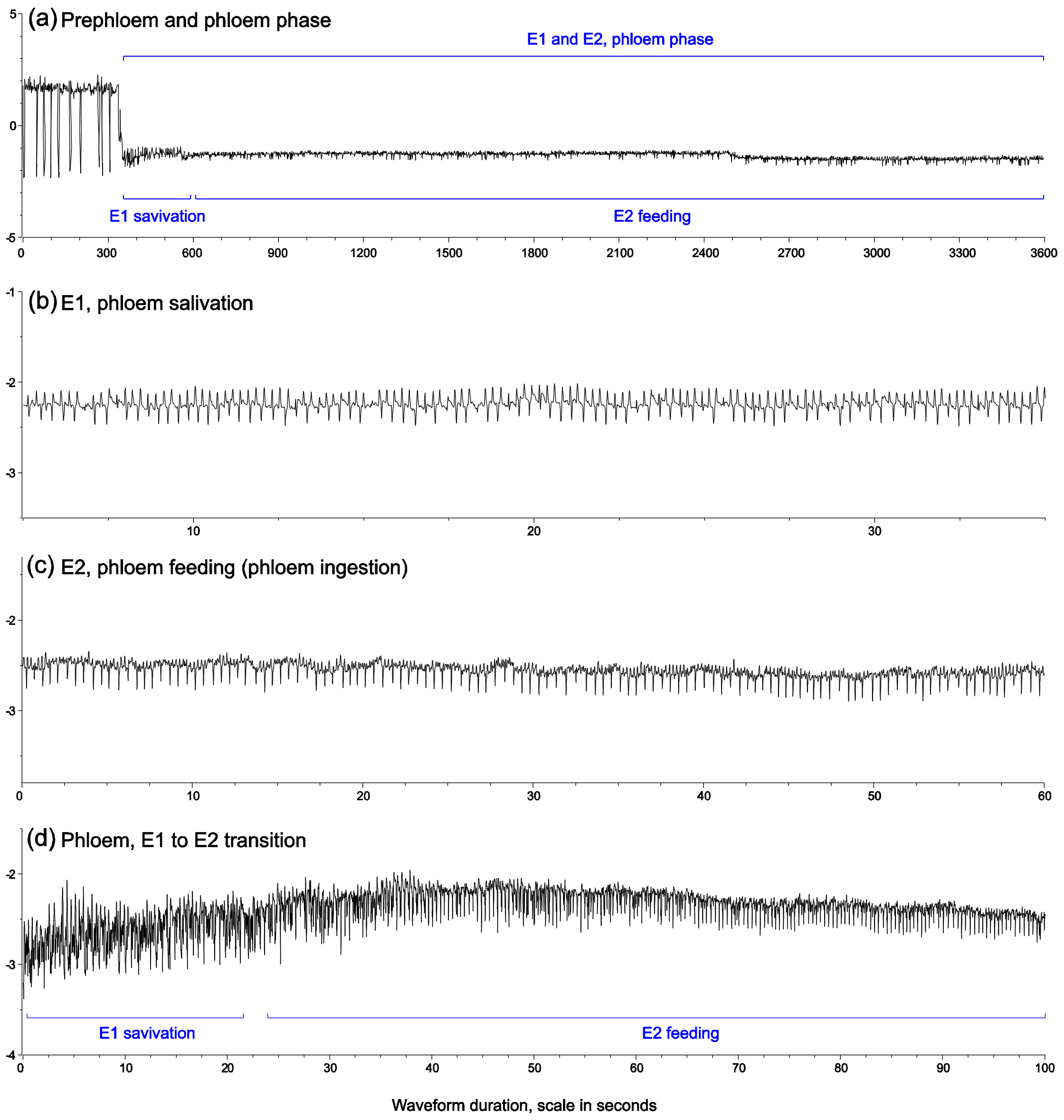

Fig. S2. Examples of waveforms representing different probing activities from EPG recordings of *R. padi* probing on wheat. (a) One hour recording on prephloem and phloem phase; in phloem phase, E1 represents phloem salivation phase and E2 represents phloem ingestion phase, (b) detailed representation of E1, phloem salivation, (c) detailed representation of E2, phloem ingestion (aphid feeding), (d) transition phase from phloem salivation (E1) to phloem ingestion (E2). Y axis represents voltage level. X axis represent waveform duration (seconds).

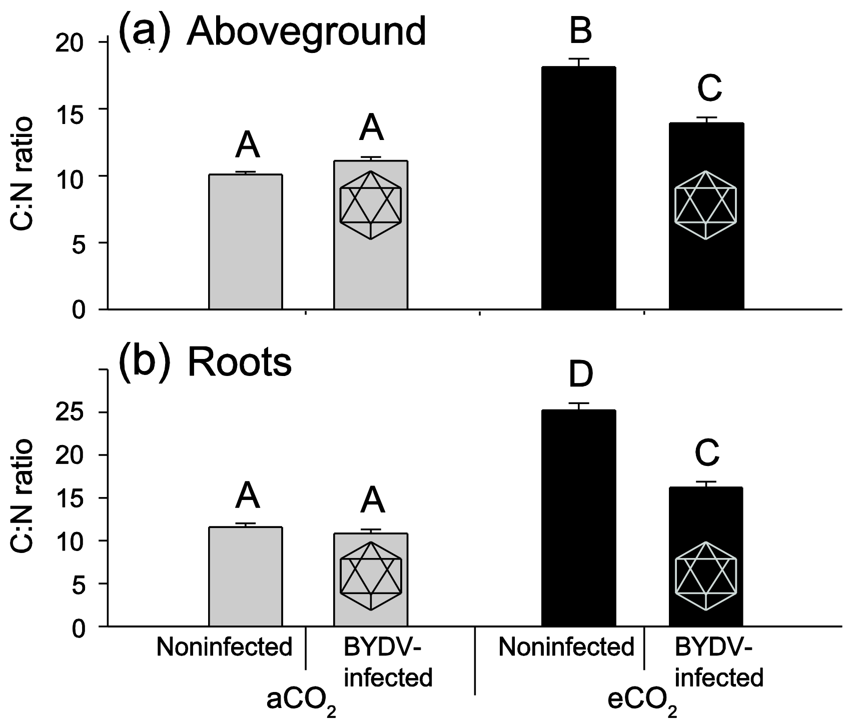

Fig. S3. Carbon to Nitrogen (C:N) ratios of (a) aboveground plant parts and (b) roots of noninfected and BYDV-infected wheat plants grown under ambient (aCO_2; 385 μmol mol-[1]) or elevated CO_2 (eCO_2; 650 μmol mol-[1]). Error bars represent standard error (SEM), different uppercase letters indicate significant differences between plant parts (aboveground and roots) and treatments (Tukey's multiple range test, $P<0.05$). Hexagon symbol indicates virus presence. The percent of N and C concentration of aboveground biomass and roots was determined by the Dumas combustion method using a CHN analyser (CHN 2000; LECO, St Joseph, MI, USA). The C:N ratio was calculated by dividing % C by % N for each plant tissue.

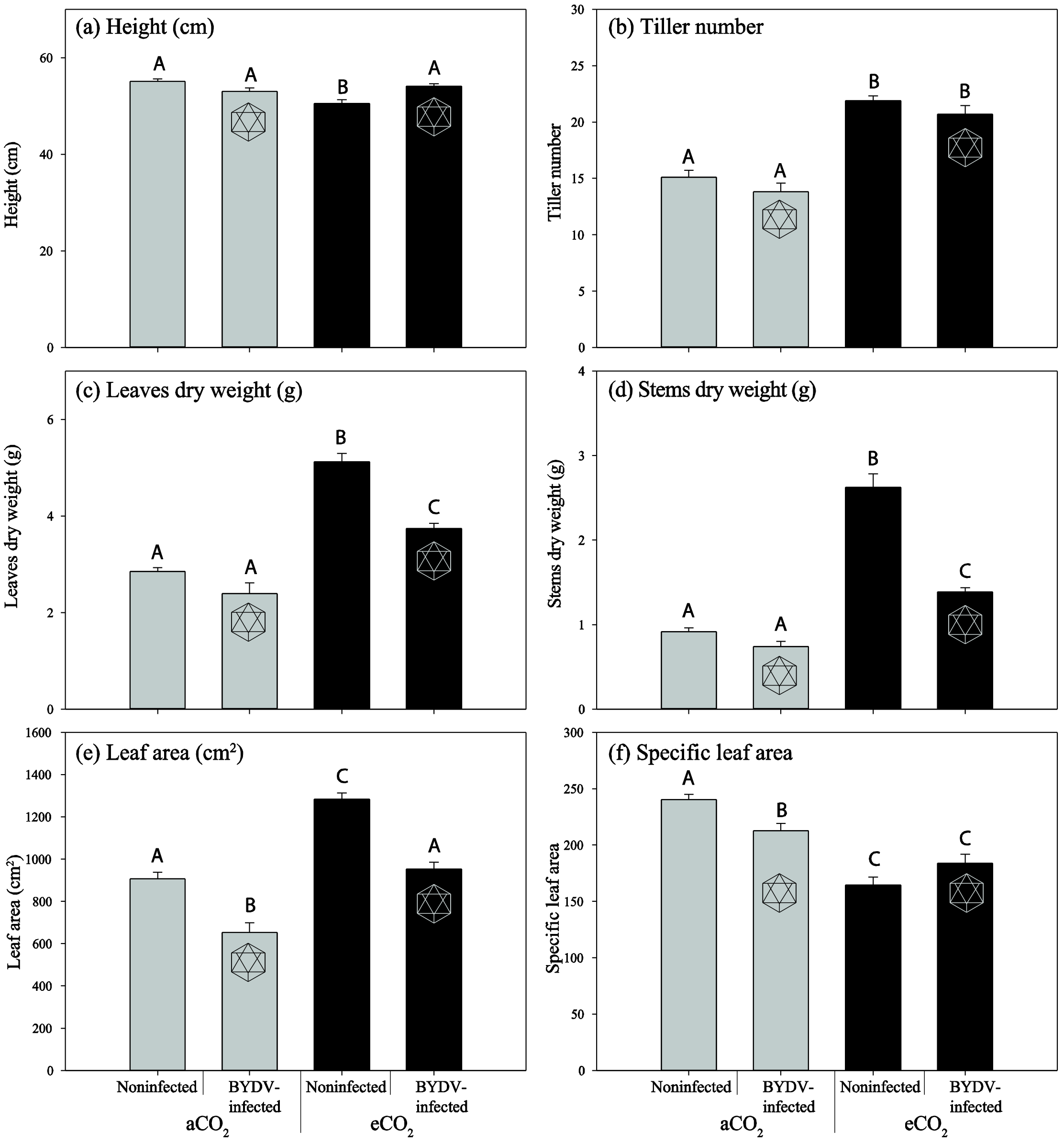

Fig. S4. (a) Height, (b) tiller number, (c) leaves dry weight (g), (d) stems dry weight (g), (e) leaf area (cm^2), and (f) specific leaf area (cm^2) of five-week-old noninfected and BYDV-infected wheat plants grown under ambient (aCO^2; 385 µmol mol-1) or elevated CO_2 (eCO_2; 650 µmol mol-^{1}). Error bars represent standard error (SEM), different uppercase letters indicate significant differences between treatments (Tukey's multiple range test, P<0.05). Hexagon symbol indicates virus presence. Leaves and stems were separated, dried at 65°C for 48 h and weighed. Leaf area was measured using a leaf area meter (LAMBDA LI-300, LiCor, NE, USA). Specific leaf area was calculated by dividing total leaf area by total leaf dry weight.
